# Characterization of Group I Metabotropic Glutamate Receptors in Rat and Human Adrenal Glands

**DOI:** 10.3389/fphys.2020.00401

**Published:** 2020-05-27

**Authors:** Ya-nan Fan, Chaohong Li, Lu Huang, Lingyun Chen, Zhao Tang, Guangye Han, Yuzhen Liu

**Affiliations:** ^1^Henan Key Laboratory of Neural Regeneration and Repairment, The First Affiliated Hospital of Xinxiang Medical University, Henan Neurology Institute, Weihui, China; ^2^Operating Room, The First Affiliated Hospital of Xinxiang Medical University, Weihui, China; ^3^Department of Urology Surgery, The First Affiliated Hospital of Xinxiang Medical University, Weihui, China

**Keywords:** metabotropic glutamate receptor, adrenal gland, catecholamine, tyrosine hydroxylase, chromaffin cell, hypoxia

## Abstract

Glutamate and its receptors have been demonstrated to promote both basal and nicotine-evoked catecholamine release in bovine chromaffin cells. Multiple glutamate receptors, including metabotropic glutamate receptors (mGluRs), are found in the adrenal glands of several species, as well as in chromaffin cells. However, there is limited information available regarding the expression of glutamate metabotropic receptor *(GRM)1-8* mRNAs and the detailed localization of group I mGluRs (mGluR1 and mGluR5) in the rat and human adrenal cortex and medulla. Therefore, we examined mRNA expression of *GRM1-8* subunits using reverse transcription-polymerase chain reaction (RT-PCR) and the distribution of mGluR1 and mGluR5 by immunostaining. The results showed that the *GRM1-8* mRNAs were expressed in both the cortex and medulla of rat and human adrenal glands with the exception of *GRM1*, which was not detectable in the rat adrenal cortex. Immunostaining of mGluR1 revealed that it was localized only in the adrenal medulla of rats but was present in both the adrenal cortex and medulla in humans. In the adrenal medulla, the central part of the adrenal glands, mGluR1 was detected in chromaffin cells but not in nerve fibers and ganglion cells. Immunoactivity of mGluR5 was visible in the capillary wall throughout the adrenal cortex and medulla in rat and human samples. Its immunoactivity was also observed in ganglion cells in the rat adrenal medulla. There was no mGluR5 immunoactivity detected in chromaffin cells and nerve fibers in the rat and human adrenal medulla. Using dissected rat adrenal medulla as a model, we found that treatment with a mGluR1 agonist activated extracellular signal-regulated kinase (ERK) 1/2 and increased the expression of tyrosine hydroxylase (TH), the rate-limiting enzyme of catecholamine synthesis. Moreover, these results showed that mGluR1 signaling was involved in hypoxia-induced upregulation of TH in the rat adrenal medulla. This study shows the expression of *GRM1-8* mRNAs in rat and human adrenal glands and indicates that glutamate, through the activation of mGluRs, may play various physiological roles in the adrenal gland. Furthermore, mGluR1 may be involved in catecholamine biosynthesis by regulating TH, and mGluR5 may affect cortical and medullar hormone levels by regulating microvascular function.

## Introduction

Glutamate is a major excitatory neurotransmitter in the central nervous system that binds to its receptors to participate in the modulation of synaptic transmission and neuronal excitability. Glutamate receptors are divided into ionotropic glutamate receptors (iGluRs) and metabotropic glutamate receptors (mGluRs). iGluRs form ion channel complexes to mediate rapid signaling and participate in excitatory synaptic transmission, neuronal plasticity, and neurotoxic processes (Cryan and Dev, 2008). mGluRs are members of the G protein-coupled receptor family; they produce slow physiological responses by regulating signal transduction cascades (Niswender and Conn, 2010). Eight mGluR subtypes (mGluR1–8) have been identified. According to the amino acid sequence homology, pharmacological characteristics, and different signal transduction mechanisms, mGluRs are divided into three groups. Group I mGluRs (mGluR1 and mGluR5) generally couple to G_*q*_/11 and activate the phospholipase C (PLC) pathway (Niswender and Conn, 2010). Group I mGluRs also activate other cascades downstream of G_*q*_, including phospholipase D, extracellular signal-regulated kinase (ERK)1/2, protein kinase B (Akt), and mammalian target of rapamycin (mTOR)/p70 S6 (Servitja et al., 1999; Ribeiro et al., 2010; Mao and Wang, 2016). Group II (mGluR2 and mGluR3) and group III (mGluR4, mGluR6, mGluR7, and mGluR8) receptors are coupled predominantly to G_*i*_ proteins, leading to the inhibition of adenylyl cyclase (Niswender and Conn, 2010).

The adrenal gland is an essential neuroendocrine organ. It is bilaterally located on top of each kidney and consists of an outer cortex and an inner medulla (Carballeira and Fishman, 1980). The adrenal cortex mainly produces cortisol and aldosterone to influence metabolism and blood pressure. The adrenal medulla is composed of highly differentiated chromaffin cells that originate from the neural crest and are homologous to sympathetic ganglion neurons (Furlan et al., 2017). The adrenal medulla predominantly produces catecholamines, including adrenaline and noradrenaline, to control the body’s response to stress (Berger et al., 2019). Activity of the adrenal medulla is regulated by direct neural input of the sympathetic cholinergic preganglionic fibers (Ganong, 1974). When sympathetic nerves are excited, preganglionic fiber ends release acetylcholine, which acts on medullary chromaffin cells and promotes the secretion and synthesis of norepinephrine and adrenaline from the adrenal medulla (Ehrhart-Bornstein and Bornstein, 2008). Tyrosine hydroxylase (TH) is the rate-limiting enzyme in the biosynthesis of catecholamine. TH transcription is regulated by multiple transcription factors, such as hypoxia-inducible factor 1α (HIF-1α), cyclic adenosine monophosphate response element-binding protein (CREB), c-fos, and jun B (Goc et al., 1992; Lim et al., 2000; Sun and Tank, 2002; Fiory et al., 2019). Thus, the biosynthesis of catecholamine is regulated not only by autonomic nerve fibers but also by non-neurogenic factors.

Accumulating evidence suggests that glutamate receptors are also present and functional in diverse peripheral non-neuronal tissues including adrenal glands (Kristensen, 1993; Wick et al., 1993; Hinoi et al., 2002; Sarría et al., 2006; Julio-Pieper et al., 2011). Using various methods, such as radioligand binding, reverse transcription-polymerase chain reaction (RT-PCR), Western blotting, immunohistochemistry, and *in situ* hybridization, several types of glutamate receptors have been detected in the adrenal glands. Subunits 2 and 3 of α-amino-3-hydroxy-5-methyl-4-isoxazolepropionic acid (AMPA) receptor iGluRs (GluR2/3), mGluR2/3, and mGluR4a are located in the rat adrenal medullary ganglion neurons, including large-sized type I and small-sized type II ganglion neurons (Sarría et al., 2006). Group I mGluRs (mGluR1 and mGluR5) have been found in cultured bovine chromaffin cells. Pharmacological experiments have indicated that mGluR1 and mGluR5 are functionally active upon the secretion of catecholamine from bovine chromaffin cells (Arce et al., 2004). iGluR and mGluR agonists can increase both basal and nicotine-evoked catecholamine release in bovine chromaffin cells (González et al., 1998).

Although previous studies indicate the expression of multiple mGluR subunits in the adrenal glands of several species, as well as in cultured bovine chromaffin cells, there is limited information regarding the expression profile of the different mGluR subtypes in the adrenal glands and the detailed localization of group I mGluRs in rat and human adrenal tissues. Here, we investigated the mRNA expression of glutamate metabotropic receptor *(GRM)1-8* subunits in the rat and human adrenal cortex and medulla and examined the localization of mGluR1 and mGluR5 in the adrenal glands. Moreover, we determined the level of ERK1/2 phosphorylation after mGluR1 stimulation in dissected adrenal medulla of rats. Finally, we tested the effect of mGluR1 on hypoxia-induced upregulation of TH protein level in dissected rat adrenal medulla.

## Materials and Methods

### Reagents and Antibodies

TRIzol reagent was purchased from Thermo Fisher Scientific (Waltham, MA, United States). The 5 × All-In-One RT MasterMix with AccuRT Genomic DNA Removal kit and 2 × PCR Taq MasterMix with dye were purchased from Applied Biological Materials (Vancouver, Canada). Rabbit anti-mGluR1, mGluR5, TH, and mouse anti-neurofilament (NF) antibodies were purchased from Abcam (Cambridge, MA, United States). Rabbit anti-p-ERK1/2, anti-ERK1/2, anti-β-actin, Alexa Fluor 488 goat anti-rabbit immunoglobulin G (IgG), Alexa Fluor 555 goat anti-mouse IgG, and anti-rabbit IgG horseradish peroxidase (HRP)-linked antibodies were purchased from Cell Signaling Technology (Danvers, MA, United States). The rabbit anti-HIF-1α antibody was purchased from Proteintech (Wuhan, China). The rabbit anti-glyceraldehyde-3-phosphate dehydrogenase (GAPDH) antibody was obtained from Boster Biological Technology (Wuhan, China). The GTVision^TM^ III Detection System/Mo & Rb Kit and the 3′-diaminobenzidine (DAB) Detection Kit were obtained from Gene Tech (Shanghai, China). Mouse anti-TH antibody and penicillin/streptomycin were ordered from Sigma (St. Louis, MO, United States). 3, 4-Dihydro-2H-pyrano[2,3-b]quinolin-7-yl)-(cis-4-methoxycyclohexyl)-methanone (JNJ 16259685) and (S)-3,5-dihydroxyphenylglycine (DHPG) were purchased from Santa Cruz Biotechnology (Dallas, TX, United States). Dulbecco’s modified Eagle media (DMEM) and the SuperSignal West Pico Chemiluminescent Substrate detection kit were purchased from Thermo Fisher Scientific (Waltham, MA, United States). Insulin was purchased from Beyotime (Shanghai, China). Fetal bovine serum (FBS) was obtained from Biological Industries (Kibbutz Beit Haemek, Israel). All primers were synthesized at the Beijing Genomics Institute (Shenzhen, China).

### Animals

Male Sprague-Dawley (SD) rats were purchased from Beijing Vital River Laboratory Animal Technology Co., Ltd. (Beijing, China). The weight of the rats at the beginning of the experiment was 230–250 g. Rats were housed in standard rodent cages with a 12-h light:12-h dark cycle and were provided food and water *ad libitum*. Animal experimental protocols were approved by the Institutional Animal Care and Use Committee of The First Affiliated Hospital of Xinxiang Medical University.

### Human Tissues

The human adrenal gland and cerebral cortex were obtained from The First Affiliated Hospital of Xinxiang Medical University (Weihui, China) with informed consent of the patients in accordance with the Declaration of Helsinki. A human adrenal gland specimen was obtained from a patient with right kidney cancer who underwent radical nephrectomy. Human cerebral cortex tissue was acquired from a patient with craniocerebral trauma. The specimens were sectioned and examined by pathologists in the hospital, and no abnormalities were found. The human study was reviewed and approved by the Institutional Review Board for Human Research of The First Affiliated Hospital of Xinxiang Medical University.

### *In situ* Experiments After Dissection of the Adrenal Medulla Following Treatment

SD rats were anesthetized with sodium pentobarbital (60 mg/kg body weight, intraperitoneal injection), and the bilateral adrenal glands were harvested using cold 0.01 M phosphate-buffered saline (PBS, pH 7.4) bubbled with 95% O_2_–5% CO_2_ gas. Next, the adrenal medulla was carefully dissociated from the adrenal gland. All dissected adrenal medullas were pooled together, and two adrenal medulla samples were randomly grouped and maintained at 37°C in a humidified atmosphere with 5% CO_2_ in a medium consisting of DMEM, 3.6 μg/ml insulin, 20% FBS, and 2% penicillin/streptomycin. After 60 min of incubation, each group of adrenal medulla samples was treated with 70 mM KCl, mGluR1 agonist, or 5% O_2_. The total number of rats allocated for each treatment depended on the specific experimental conditions listed below.

### RNA Extraction and Semiquantitative RT-PCR

Total RNA was extracted using the TRIzol reagent (Thermo Fisher Scientific, Waltham, MA, United States) according to the manufacturer’s instructions. cDNA was synthesized through RT using 1 μg of total RNA, 4 μl of 5 × All-In-One RT MasterMix with AccuRT Genomic DNA Removal kit in a total reaction volume of 20 μl, and the following incubation conditions: 25°C for 10 min, 42°C for 50 min, and 85°C for 5 min. For PCR, 2 μl of cDNA was amplified using 2 × PCR Taq MasterMix with dye in a total reaction volume of 50 μl. The PCR conditions consisted of pre-denaturation at 94°C for 3 min, followed by repetitive cycles (see Tables 1, 2 for specific details) with denaturation at 94°C for 30 s, annealing at primer-specific temperatures for 30 s, and extension at 72°C for 1 min. The last cycle was followed by an extension step at 72°C for 5 min. The PCR products were separated using electrophoresis on a 1.2% agarose gel and analyzed by a gel imaging system. The housekeeping genes β*-actin* for rat samples and *GAPDH* for human samples were used as loading controls. Rat or human brain cDNA was used as positive controls. RNA without reverse transcriptase was used in the RT reaction to obtain a negative cDNA control, which was used as the template for the negative PCR control. A total of three biological replicates were conducted for each group. All primers were designed using Primer-BLAST^1^. The sequences of the primers with their respective annealing temperatures, PCR cycles, and lengths of PCR products are summarized in Tables 1, 2.

**TABLE 1:** Sequences of rat primers and PCR conditions.

Gene	Primer sequence	PCR cycles	Annealing Tm (°C)	Length (bp)
*GRM1*	F: 5′-GGCTGGTATTTTCCTCGGCT-3′ R: 5′-GTGTGGGGGTTTTCAAAGCTG-3′	40	57	829
*GRM2*	F: 5′-CTCCTCACCAAGACCAATCG-3′ R: 5′-GTGGTTACAGCGCAATGTCA-3′	42	52	222
*GRM3*	F: 5′-TATTCTCAGTCCTCTGCAAG-3′ R: 5′-TTGTAGCACATCACTACATACC-3′	42	50	261
*GRM4*	F: 5′-ACAGTCAGCCGACAAGCTGTACAT-3′ R: 5′-ATGGTTGGTGTAGGTGACGTAGGT-3′	42	57	304
*GRM5*	F: 5′-GGCATTATCGTGGCCCTCTT-3′ R: 5′-TTTTCCGTTGGAGCTTAGGGT-3′	40	56	528
*GRM6*	F: 5′-CTGTTCCGCTCTTCCTCACTTG-3′ R: 5′-ATTCAGACCTTGGCTCACCGAC-3′	42	55	228
*GRM7*	F: 5′-ACAACTGGCGATCACTTCCA-3′ R: 5′-GTTCATGGTCTTATGCTCATC-3′	42	50	115
*GRM8*	F: 5′-AAACAAACCGTATCCACCG-3′ R: 5′-ATCCCAGGGAACAAATGAGT-3′	42	53	258
*β-actin*	F: 5′-GGGAAATCGTGCGTGACATT-3′ R: 5′-CGGATGTCAACGTCACACTT-3′	35	53	253

**TABLE 2:** Sequences of human primers and PCR conditions.

Gene	Primer sequence	PCR cycles	Annealing Tm (°C)	Length (bp)
*GRM1*	F: 5′-TCATATGCAGCTCGGATGGAC-3′ R: 5′-GGCTGAGAAATCAGCAGCTTCA-3′	40	57	601
*GRM2*	F: 5′-CTATGGCGAGACAGGCATTGA-3′ R: 5′-CATCCTCAGAACGGGTGAACA-3′	42	55	179
*GRM3*	F: 5′-GCACCTCAACAGGTTCAGTGT-3′ R: 5′-TGGTGGAGTCGAGGACTTCC-3′	42	55	110
*GRM4*	F: 5′-AATACCAGCTGCGCAACGAT-3′ R: 5′-CTGTCTTCTTCCGCTCACCC-3′	42	55	162
*GRM5*	F: 5′-GAGCGGAGGGAATGAGCTTG-3′ R: 5′-TGCCATACTGTTCACGGACC-3′	40	57	541
*GRM6*	F: 5′-TCCAGACAACCACGCTAACC-3′ R: 5′-GGGAGGAAATCTCCCGCAAA-3′	42	56	544
*GRM7*	F: 5′-TCCTTGCTGTTGGACCTGTGAG-3′ R: 5′-CATTGTAGCGGATGAAAGTGGC-3′	40	57	233
*GRM8*	F: 5′-CCAGAGCTAAGTGATAACACCAG-3′ R: 5′-TCTGTGACTGAGCAATGCAAA-3′	40	57	214
*GAPDH*	F: 5′-GCAGGGGGGAGCCAAAAGGGT-3′ R: 5′-TGGGTGGCAGTGATGGCATGG-3′	30	55	219

### Immunohistochemistry

After transcardial perfusion fixation with 4% neutral formaldehyde, the bilateral adrenal glands were removed, and immunohistochemical staining was performed on 3-μm-thick tissue sections. Adrenal gland sections were deparaffinized, washed three times with PBS, and immersed in 0.01 M citrate buffer (pH 6.0); antigen retrieval was performed at 95°C for 15 min. A solution of 3% H_2_O_2_ was used to block endogenous peroxidase followed by washing with PBS three times. PBS containing 0.2% Triton X-100 was used to permeate the tissue for 15 min, and non-specific tissue sites were blocked with 10% goat serum for 1 h at room temperature. Then, primary antibodies, including anti-mGluR1 (1:200, ab82211, Abcam) and anti-mGluR5 antibodies (1:200, ab76316, Abcam), were added to the sections, followed by incubation overnight at 4°C. Negative controls were prepared by replacing the primary antibody with PBS. According to the instructions provided in the GTVision^TM^ III Detection System/Mo & Rb Kit, the sections were treated with an anti-rabbit/mouse HRP-labeled secondary antibody (GK500705, Gene Tech) for 30 min at 37°C and then reacted with DAB and stained for further microscopic analysis. Finally, the sections were stained with hematoxylin for 2 min and dehydrated. The sections were then observed under a light microscope and photographed.

### Immunofluorescence

A double-staining immunofluorescence assay was used to identify the localization of mGluR1 and mGluR5 in rat and human adrenal glands. Primary antibody dilutions with 5% bovine serum albumin were prepared, and a simultaneous incubation of the adrenal sections with monoclonal rabbit anti-mGluR1 and mGluR5 antibodies (1:200) and monoclonal mouse anti-TH antibody (1:2,000, T2928, Sigma) was performed. Next, the sections were incubated simultaneously with each of the above mentioned monoclonal antibodies for the glutamate receptors and a monoclonal mouse anti-NF antibody (1:100, ab7794, Abcam) overnight at 4°C. The following secondary antibodies were used: Alexa Fluro 488 goat anti-rabbit IgG (1:300, 4412S, CST) and Alexa Fluro 555 goat anti-mouse IgG (1:300, 4409S, CST). The staining was examined with an Axio Observer A1 microscope and photographed with an AxioCam MRc5 camera (Carl Zeiss, Göttingen, Germany).

### Western Blotting

After treatment, dissected rat adrenal medulla sections were harvested and lysed with RIPA lysate buffer containing 1% protease inhibitor. Twenty-five micrograms of total protein lysate was separated by 10% sodium dodecyl sulfate-polyacrylamide gel electrophoresis, and the bands were transferred onto a nitrocellulose membrane. The blots were blocked with 5% non-fat dry milk and probed overnight at 4°C with primary antibodies against p-ERK1/2 (4370, CST), ERK1/2 (4695, CST), HIF-1α (20960-1-AP, Proteintech), TH (ab75875, Abcam), GAPDH (BA2913, Boster), and β-actin (8457S, CST) (all diluted by 1:1,000). The membranes were washed three times with Tris-buffered saline-Tween 20 and incubated with anti-rabbit IgG HRP-linked antibody (1:8,000, 7074, CST) at room temperature for 1 h. After washing, the membrane was immersed in SuperSignal West Pico Chemiluminescent Substrate, and immunoreactivity was visualized with the Amersham Imager 600 system. ImageJ software was used to quantify the optical density of each band.

### Statistical Analysis

Data are presented as the means ± standard deviations (SDs) of results obtained from three separate experiments. Statistical evaluation of the data was performed using one-way ANOVA. The experimental data were consistent with the homogeneity of variance, and least significant difference (LSD) *t*-test and Student–Newman–Keuls (SNK)-*q* test were performed for a *post-hoc* intergroup comparison. ^∗^*P* < 0.05 was considered statistically significant.

## Results

### mRNA Expression of GRMs in the Rat and Human Adrenal Cortex and Medulla

**FIGURE 1:**
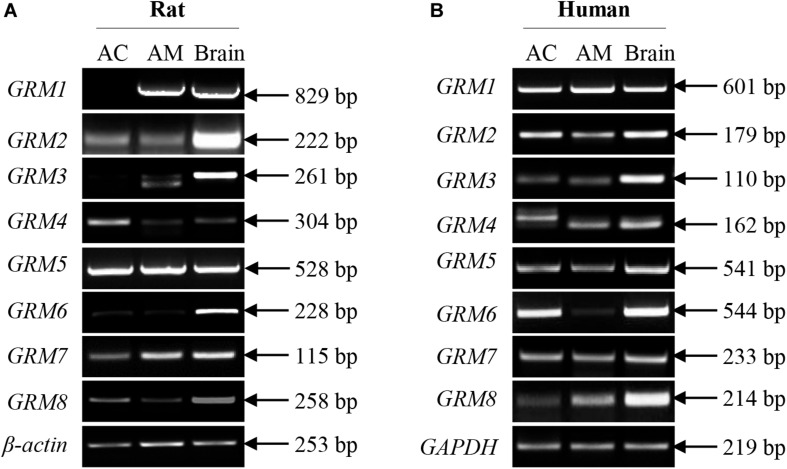
mRNA expression of all *GRM* subunits in rat and human adrenal glands. Representative results of RT-PCR showing the mRNA expression patterns of *GRM1-8* subunits in the adrenal cortex and adrenal medulla of rats **(A)** and humans **(B)**. RNA samples from rat and human brain cerebral cortices are used as positive controls (AC, adrenal cortex; AM, adrenal medulla).

Although some mGluR subunits have been demonstrated to be present in the adrenal glands, the expression of mGluR subunits in the adrenal cortex and medulla remains undefined. We performed RT-PCR analysis to detect the mRNA expression of *GRM1-8* subunits in the rat and human adrenal cortex and medulla. Figure 1A showed that the transcripts of *GRM1-8* subunits were present in the rat adrenal cortex and medulla, except for *GRM1* in the rat adrenal cortex. Among these *GRM* subunits, PCR product bands for *GRM6* in rat cortex and medulla were very faint, and PCR product for *GRM3* in rat adrenal medulla showed a double band, which was smaller in size than that in rat brain positive control (Figure 1A). All *GRM* subunit mRNAs were detected in the human adrenal cortex and medulla (Figure 1B). *GRM6* in the human adrenal medulla showed as a very faint PCR product band, and the PCR band for *GRM4* in the human adrenal cortex showed a difference in size compared to adrenal medulla and brain positive control (Figure 1B).

### Protein Expression and Distribution of mGluR1 and mGluR5 in Rat and Human Adrenal Cortex and Medulla Sections

**FIGURE 2:**
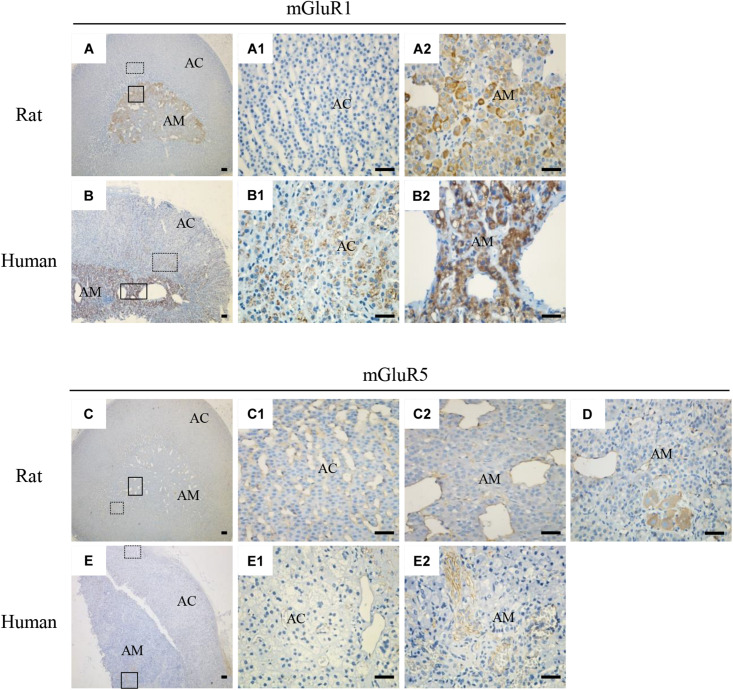
Immunohistochemical staining of mGluR1 and mGluR5 in rat and human adrenal glands. Representative images of rat and human adrenal sections immunostained with antibodies against mGluR1 and mGluR5. Detailed staining images of the dotted and solid line-framed areas **(A–C,E)** are shown with higher magnification in **(A1,A2,B1,B2,C1,C2,E1,E2)**. **(D)** shows mGluR5 immunoreactivity present in the cytoplasm of ganglion cells in the rat adrenal medulla (AC, adrenal cortex; AM, adrenal medulla). Scale bar = 50 μm.

To explore the localization of mGluRs in the adrenal glands, immunohistochemistry was performed using antibodies for the group I mGluRs (mGluR1 and mGluR5). As shown in Figure 2, mGluR1 immunoreactive intensity in the rat (Figures 2A,A2) and human adrenal medulla (Figures 2B,B2) was much stronger than that in the adrenal cortex (Figures 2A1,B1). Within the stained adrenal medulla, the intensity of mGluR1-specific staining varied among the cells. mGluR1 immunoreactivity in the adrenal cortex was not observed in the rat samples, but was visible in the human sections, with the highest intensity being observed in the innermost region of the cortex; the intensity decreased gradually toward the outer region. mGluR5-specific immunohistochemical staining was found within the whole adrenal medullar and cortex areas of both the rat and human sections (Figures 2C,E). mGluR5 staining was mainly observed in capillary endothelial cells in the adrenal cortex and medulla (Figures 2C1,C2,E1,E2). Notably, mGluR5 staining was stronger in the rat adrenal cortex and medulla and human adrenal medulla but weaker in the human adrenal cortex (Figure 2E1). A few ganglion cells positively labeled with mGluR5 immunoreactivity were also observed in the rat adrenal medulla (Figure 2D).

### Cellular Localization of mGluR1 and mGluR5 in Rat and Human Adrenal Medulla Sections

**FIGURE 3:**
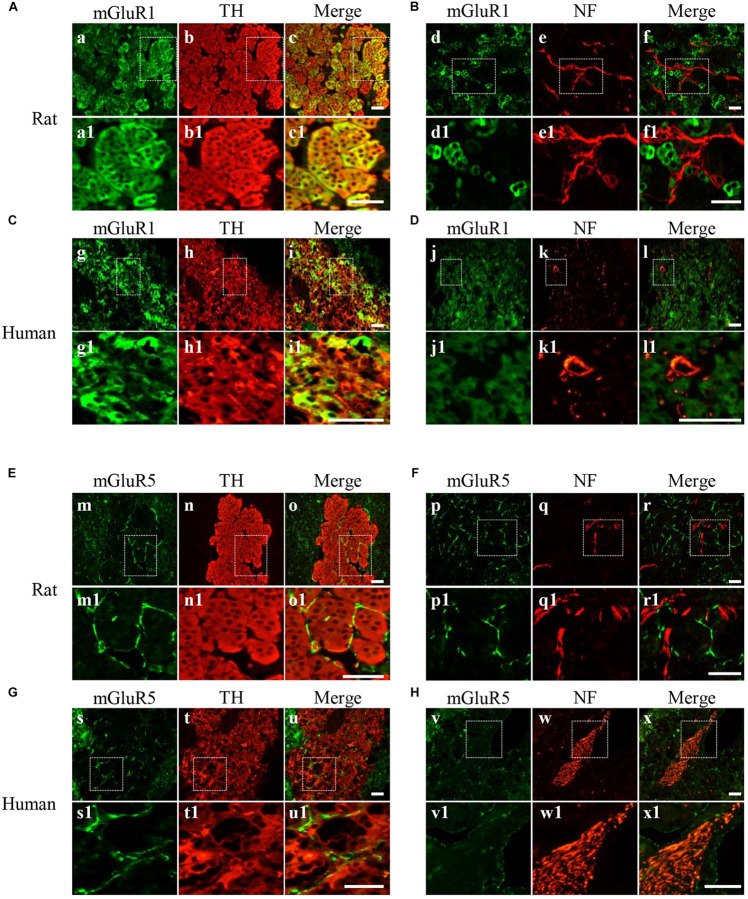
Cellular localization of mGluR1 and mGluR5 in rat and human adrenal medulla. **(A,C)** Double immunofluorescence staining of mGluR1 (green) with tyrosine hydroxylase (TH) (red) in rat and human adrenal medulla **(a–c1,g–i1)**. TH-labeled catecholaminergic cells. **(B,D)** Double immunofluorescence staining of mGluR1 (green) with neurofilament (NF) (red) in rat and human adrenal medulla **(d–f1,j–l1)**. NF-labeled nerve fibers and neurons. **(E,G)** Double immunofluorescence staining of mGluR5 (green) with TH (red) in rat and human adrenal medulla **(m–o1,s–u1)**. **(F,H)** Double immunofluorescence staining of mGluR5 (green) with NF (red) in rat and human adrenal medulla **(p–r1,v–x1)**. Higher magnification images of framed areas are shown in matched lower panels. Scale bar = 25 μm.

For detailed investigation of the distribution of mGluR1 and mGluR5 in the adrenal medulla, double immunofluorescence was performed. mGluR1-immunoreactive signals with varying intensity were found in TH-labeled cells (Figures 3A,C) but were not co-localized with NF-positive nerve fibers and ganglion cells (Figures 3B,D) in the adrenal medulla, indicating that mGluR1 was present in the chromaffin cells but not in the nerve fibers and ganglion cells in the rat and human adrenal medulla. mGluR5 staining showed a stripe-like pattern and was distributed in the blood sinus endothelium but not on nerve bundles with multiple NF-positive nerve fibers (Figures 3E–H). The detected immunoreactive signals did not merge with either TH immunoreactivity (Figures 3E,G) or NF immunoreactivity (Figures 3F,H), indicating that mGluR5 is not present in chromaffin cells, as well as in the nerve fibers in the rat and human adrenal medulla.

### Analysis of Responses to the Stimulation of mGluR1 and mGluR5 After Dissection of the Adrenal Medulla *in situ*

**FIGURE 4:**
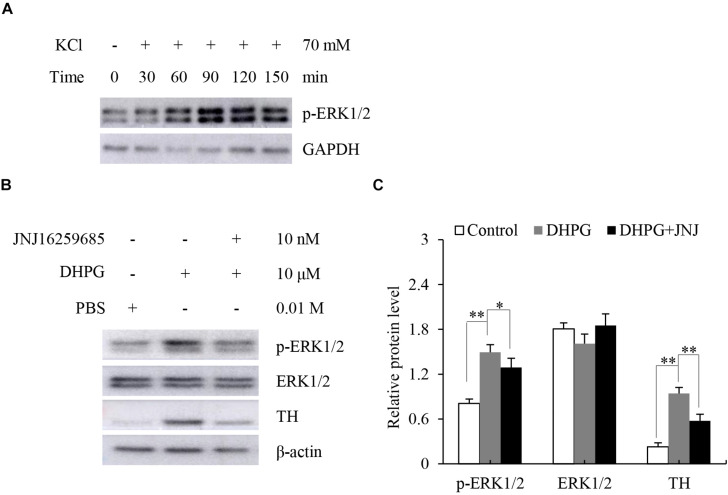
Response of dissected rat adrenal medulla to KCl and a mGluR1 agonist. **(A)** Western blotting analysis of phospho-extracellular signal-regulated kinase 1/2 (p-ERK1/2) in dissected rat adrenal medullas treated with 70 mM KCl at the indicated time. Glyceraldehyde-3-phosphate dehydrogenase (GAPDH) is used as an internal control. **(B)** Western blotting of p-ERK1/2, ERK1/2, and tyrosine hydroxylase (TH) in dissected rat adrenal medullas treated with 10 μM (S)-3,5-dihydroxyphenylglycine (DHPG) for 60 min in the absence or presence of 10 nM 3, 4-dihydro-2H-pyrano[2,3-b]quinolin-7-yl)-(cis-4-methoxycyclohexyl)- methanone (JNJ16259685). PBS treatment is the vehicle control. β-actin is used as an internal control. **(C)** Quantification of the relative protein levels of p-ERK1/2, ERK1/2, and TH. The β-actin level is used to normalize the levels of these proteins. Data from three biological triplicates are presented as the mean ± SD. ^∗^*P* < 0.05 and ^∗∗^*P* < 0.01 vs. the DHPG group.

Stimulation of mGluR1 and mGluR5 leads to the activation of the protein kinases ERK1/2. To further verify mGluR1 and mGluR5 functional activity in the adrenal medulla, we dissected the rat adrenal medulla, which is known to serve as an appropriate study model between *in vivo* animal and cell culture models to investigate the function of the adrenal medulla *in vitro* (Fujinaga et al., 1999). It has been reported that KCl treatment causes activation of ERK1/2 in chromaffin cells (Cox and Parsons, 1997). To determine the proper function of our model *in vitro*, the dissected adrenal medulla sections were treated with KCl, and Western blotting analysis was subsequently performed. As shown in Figure 4A, ERK1/2 was phosphorylated in a time-dependent manner after KCl treatment. Maximal phosphorylation was obtained 90 min after treatment, which then slowly declined at 120 min. These results indicate that our model is suitable for subsequent experiments.

Since mGluR1 is mainly located in the adrenal medullary chromaffin cells (Arce et al., 2004), we tested the response of mGluR1 in the adrenal medulla by treating the dissected adrenal medulla sections with DHPG, a non-specific agonist of mGluR1, for 60 min. Next, we performed Western blotting analysis to examine the phosphorylation of ERK1/2 and the expression levels of TH, which is the rate-limiting enzyme of catecholamine synthesis. Treatment with DHPG resulted in the activation of ERK1/2 compared to the control group. Pretreatment with JNJ16259685, a specific mGluR1 antagonist, blocked the phosphorylation of ERK1/2 induced by DHPG. Interestingly, DHPG also increased protein levels of TH, whereas JNJ16259685 blocked this effect (Figures 4B,C). Thus, these observations suggest that mGluR1 is functional in the adrenal medulla, and mGluR1 may increase catecholamine synthesis by the upregulation of TH.

### Effect of Hypoxia on the Expression of Tyrosine Hydroxylase in Dissected Rat Adrenal Medulla

**FIGURE 5:**
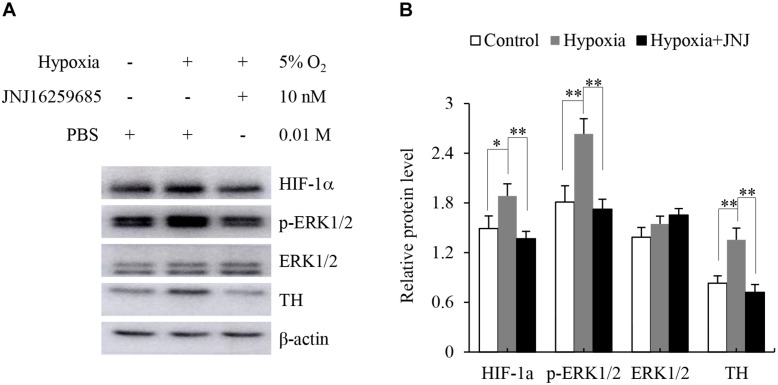
Effect of mGluR1 on tyrosine hydroxylase (TH) protein levels under hypoxic conditions in dissected rat adrenal medulla. **(A)** Western blotting analysis of hypoxia-inducible factor 1α (HIF-1α), phospho-extracellular signal-regulated kinase 1/2 (p-ERK1/2), ERK1/2, and TH of dissected rat adrenal medulla. The dissected tissues were stored at 37°C in a 5% CO_2_ incubator with 5% O_2_ (hypoxia) for 5 h with or without JNJ16259685 (10 nM) pretreatment for 30 min. An incubator with 21% O_2_ was used as a normoxic control, and PBS treatment served as the vehicle control. β-actin is used as an internal control. **(B)** Quantification of the relative levels of HIF-1α, p-ERK1/2, ERK1/2, and TH. The β-actin level is used to normalize the levels of these proteins. Data from three biological triplicates are presented as the mean ± SD. ^∗^*P* < 0.05 and ^∗∗^*P* < 0.01 vs. the hypoxia group.

The adrenal medulla plays a crucial role in the physiological responses to stressors, such as hypoxia. Previous studies have indicated that hypoxia enhances the expression of TH (Schmitt et al., 1992; Pépin et al., 1996). Therefore, we explored whether activation of mGluR1 was involved in the hypoxia-induced expression of TH in the adrenal medulla. To this end, rat adrenal medulla samples were subjected to treatment with 5% O_2_ for 5 h in the absence or presence of JNJ16259685. As shown in Figure 5, 5% O_2_ treatment distinctly promoted the phosphorylation of ERK1/2 and increased the protein levels of HIF-1α and TH compared with those in the control group; inhibition of mGluR1 with JNJ16259685 blocks these effects. These data indicate that mGluR1 signaling is involved in hypoxia-induced catecholamine synthesis.

## Discussion

The major findings of this study were the following: (1) mRNA expression of *GRM1-8* subunits was detected in the rat and human adrenal cortex and medulla; (2) the presence of mGluR1 was detected in the human adrenal cortex and medulla, whereas mGluR1 was found only in the rat adrenal medulla. mGluR5 immunoactivity was scattered throughout the adrenal medulla and cortex in the rat and human sections. In the adrenal medulla, mGluR1 localized to chromaffin cells but not nerve fibers and ganglion cells. mGluR5 immunoactivity was detected in the adrenal capillary endothelial cells and ganglion cells but not in chromaffin cells and nerve fibers; (3) activation of mGluR1 promoted the phosphorylation of ERK1/2 and increased the protein levels of TH, and mGluR1 signaling was involved in hypoxia-induced upregulation of TH in the dissected rat adrenal medulla.

Previous reports have shown some inconsistencies in the expression of mGluR subunits in the adrenal glands. For example, Scaccianoce et al. (2003) reported that among the *GRM1-8* subunits, only *GRM5* and *GRM7* mRNAs are detected in rat and mouse adrenal glands. However, Sarría et al. (2006) found that mGluR2/3 and mGluR4 immunoreactivities are observed in the rat adrenal gland. To further clarify these discrepancies, we designed our study to provide a comprehensive analysis of the mRNA expression of the subunits *GRM1-8* in the cortex and medulla of rat and human adrenal glands. We found that all *GRM1-8* mRNAs, except for *GRM1*, which was not detected in the rat adrenal cortex, were expressed in both the cortex and medulla of the rat adrenal gland (Figure 1A). Moreover, we found that mRNAs of all *GRM1-8* subunits were expressed in the cortex and medulla of the human adrenal gland (Figure 1B), suggesting that the expression of *GRM1-8* mRNAs in adrenal glands may vary across species. As shown in Figure 1, all amplicons produced using either the rat or human brain cDNA templates were detected with the expected lengths, indicating that these primers are specific for the identification of each mRNA of rat and human *GRM1-8*. Although the sizes of most PCR products for the *GRM*s in the adrenal cortex or medulla were identical to that of the brain positive control, the PCR amplicon sizes of some *GRMs*, including rat *GRM3* and human *GRM4*, were slightly different compared to the respective expected sizes. These variations might result from gene-specific alternative splicing; however, further studies should be done to confirm this hypothesis. To the best of our knowledge, this is the first study to report the expression of the *GRM1-8* subunits in human adrenal glands.

The adrenal gland is known to be composed of the cortex, medulla, blood vessels, nerves, and connective tissue. The adrenal medulla contains chromaffin cells, which produce catecholamines, including epinephrine and norepinephrine, and a small population of ganglion cells. Although it has been demonstrated using immunocytochemistry that mGluR1 and mGluR5 are present in cultured bovine chromaffin cells (Arce et al., 2004), the distribution of both mGluRs in the adrenal gland is unclear.

Using immunostaining, we observed mGluR5 immunoactivity in ganglion cells (Figure 2D), as well as in capillary endothelial cells (Figures 2C–C2,E–E2), but not in TH-positive chromaffin cells and NF-positive nerve fibers in the medulla (Figures 3E–H), inconsistent with previous findings from *in vitro* cell culture. For example, Arce et al. (2004) reported that both chromaffin cells and cortical neurons express both mGluR1α and mGluR5, suggesting that there may be differences between cultured cells and intact organs or species. There is minimal information on mGluR5 expression in ganglion cells of the adrenal medulla. It is known that ganglion neurons in the adrenal medulla innervate chromaffin cells to control catecholamine release (Afework et al., 1994). Therefore, we speculated that the function of mGluR5 in the ganglion may be involved in the control of hormone release. However, Pokusa et al. (2014) reported that blocking mGluR5 cannot restrain stress-induced catecholamine release in rats, indicating that mGluR5 in the adrenal medulla is not involved in the secretion of epinephrine and norepinephrine. Therefore, the function of mGluR5 in the adrenal ganglion remains to be investigated. Previous studies have reported the expression of mGluR5 in rat meningeal microvasculature (Gillard et al., 2003) and human vascular endothelial cells (Collard et al., 2002) and the role of mGluR5 in microvascular permeability in endothelial cells (Collard et al., 2002). Interestingly, in this study, mGluR5 immunostaining was also observed in the capillary wall throughout the adrenal cortex and medulla. Thus, mGluR5 expression in the vascular endothelium may indirectly affect cortical and medullar hormone levels by regulating vascular permeability, as it has been reported that the blockade of mGluR5 increase restraint stress-induced corticosterone release (Dubovicky and Jezova, 2004; Pokusa et al., 2014).

In this study, the immunohistochemical staining showed that mGluR1 was detected in the human adrenal medulla and cortex, as well as in the rat adrenal medulla, rather than in the rat adrenal cortex (Figures 2A–A2,B–B2), which is consistent with the results obtained by RT-PCR. Within the medulla, mGluR1 was enriched in TH-positive chromaffin cells (Figures 3A,C). It has been reported that DHPG, an agonist of group I mGluRs, results in the secretion of catecholamine from cultured bovine chromaffin cells (Arce et al., 2004). Accordingly, our data provide clear evidence that mGluR1 in chromaffin cells may play a role in the adrenal medulla during the secretion of catecholamines.

Group I mGluRs, as G protein-coupled receptors, activate multiple signaling pathways, including ERK1/2 signaling. It has been noted that phosphorylation of ERK1/2 activates TH to promote catecholamine synthesis (Haycock et al., 1992). To identify whether mGluR1 regulated catecholamine synthesis in the adrenal medulla, we dissected the rat adrenal medulla to generate a model between the cell culture and animal *in vivo* models to study the function of mGluR1. Interestingly, we found that selective inhibition of mGluR1 blocked the phosphorylation of ERK1/2 as well as upregulation of TH induced by DHPG-mediated activation of group I mGluRs. Our data demonstrated that mGluR1 was linked to catecholamine synthesis by activation of ERK1/2 in the adrenal medulla. The sympathetic-adrenal medullary system plays a key role in the physiological response of an organism to a series of stressors such as physical, chemical, and harsh environmental stresses (Marshall, 1994). Hypoxia, as a source of environmental stress, not only directly increases sympathetic nerve activity but also affects the adrenal medulla to regulate catecholamine secretion and synthesis (Kumar et al., 2015). Chromaffin cells can directly sense hypoxia through the activation of the transcription factor HIF-1α (Peng et al., 2006). As TH is a target gene of HIF-1α, hypoxia enhances the synthesis of catecholamine by activating transcription of TH (Fiory et al., 2019). In the present dissected rat adrenal medulla model, as expected, we found that hypoxia treatment upregulated HIF-1α and TH protein levels. Moreover, blocking mGluR1 effectively prevented hypoxia-induced upregulation of TH levels, suggesting that mGluR1 may contribute to catecholamine biosynthesis under hypoxic conditions.

One limitation of this study is that physiological evidence favoring the mGluR1-dependent glutamatergic transduction contributing to catecholamine synthesis and/or release in response to stress, such as hypoxia, is not provided. It is critical to further identify such roles of mGluR1 in rat adrenal glands *in vivo*.

In summary, we present the mRNA expression profiles of the *GRM1-8* subunits in the rat and human adrenal cortex and medulla. Moreover, we showed that mGluR1 was mainly present in adrenal medulla chromaffin cells, and mGluR5 was expressed in capillary endothelium and ganglion cells. We provided evidence that mGluR1 might increase catecholamine synthesis by activation of ERK1/2, and the mGluR1 signaling pathway was likely to play a significant role in hypoxia-induced catecholamine synthesis. In addition, mGluR5 might affect cortical and medullar hormone levels by regulating microvascular function. These findings suggest that mGluR1- and mGluR5-dependent glutamatergic signaling pathways are functional in the adrenal glands and may be involved in the response of these glands to stress, especially hypoxia.

## Data availability statement

The raw data supporting the conclusions of this article will be made available by the authors, without undue reservation, to any qualified researcher.

## Ethics Statement

The studies involving human participants were reviewed and approved by Institutional Review Board for Human Research of The First Affiliated Hospital of Xinxiang Medical University, China. The patients/participants provided their written informed consent to participate in this study. The animal study was reviewed and approved by Institutional Animal Care and Use Committee of The First Affiliated Hospital of Xinxiang Medical University, China.

## Author Contributions

YF, CL, LH, and YL contributed to the conception and design, collection and assembly of data. YF, CL, and YL contributed to the data analysis and interpretation. YF and YL contributed to the manuscript writing. YF, CL, LH, LC, ZT, GH, and YL contributed to the final approval of the manuscript. LC, ZT, and GH contributed to the provision of study materials. YL contributed to the supervision of the entire study. All authors have read and approved the final manuscript.

## Conflict of Interest

The authors declare that the research was conducted in the absence of any commercial or financial relationships that could be construed as a potential conflict of interest.

## Footnotes

1 https://www.ncbi.nlm.nih.gov/
